# Complete deletion of the *Chlamydia muridarum* putative cytotoxin locus reveals contributions during invasion in tissue culture and oviduct pathology during murine genital tract infection

**DOI:** 10.1128/iai.00419-25

**Published:** 2025-09-22

**Authors:** Lucie H. Berclaz, Gracie Eicher, Grace Wieselquist, Akosua Frimpong, Aria Mallare, Rebeccah S. Lijek, Kenneth A. Fields

**Affiliations:** 1 Department of Biological Sciences, Mount Holyoke College 7397, South Hadley, Massachusetts, USA; 2 Department of Microbiology, Immunology, & Molecular Genetics, University of Kentucky College of Medicine 12252, Lexington, Kentucky, USA; University of California Davis, Davis, California, USA

**Keywords:** pathogenesis, mutagenesis, large clostridial toxin

## Abstract

Chlamydiaceae is a family of obligate intracellular bacteria that infect a wide range of human and animal hosts. *Chlamydia muridarum* is a murine-specific species that has been leveraged as an efficacious model of disease mediated by human-specific *Chlamydia trachomatis*. Genes within the plasticity zone, a region of the chromosome with increased genetic variation across species and serovars, are speculated to contribute to species-specific pathogenesis. *C. muridarum* expresses three homologous proteins (TC0437–0439) that show similarity to large clostridial cytotoxins. The putative chlamydial cytotoxins have been proposed to mediate immediate toxicity in highly infected epithelial cells by interfering with actin polymerization. We utilized FRAEM mutagenesis to delete all three putative cytotoxins (*tc0437–0439*). The null strain retained immediate cytotoxicity but exhibited decreased invasion efficiency in tissue culture. During murine infections of the female genital tract, the absence of the putative cytotoxins caused decreased oviduct pathology and did not impact bacterial burden in the upper genital tract. These results indicate that the putative cytotoxins contribute to infection at the cellular level and in the female genital tract of mice.

## INTRODUCTION

Members of the Chlamydiaceae family are successful obligate intracellular parasites of both animals and humans. All *Chlamydia* spp. can infect mucosal epithelial cells, exhibit a biphasic developmental cycle alternating between infectious elementary bodies (EBs) and vegetative reticulate bodies (RBs), and develop within a vacuole termed an inclusion (1). Despite conserved biology and genome content, these bacteria have evolved to infect a wide variety of hosts (2). Economically impactful animal pathogens include *C. suis*, *C. abortus*, *C. pecorum,* and *C. psittaci* (3). *C. trachomatis* (*Ctr*) is a human pathogen where serovars A–C are ocular-specific pathogens causing blindness and urogenital-specific *Ctr* serovars D–K are agents of human sexually transmitted disease (4). Genital infections can result in serious disease sequelae that negatively impact female reproductive health (5) where chronic inflammation is regarded as a leading contributor to fibrosis and scarring in the upper genital tract of females (6–8). *Ctr* serovars L1–L3 represent additional sexually transmitted pathogens, but bacteria replicate primarily within regional lymphatics and infections manifest as ulcerative lymphogranuloma venereum (LGV) (9). *C. muridarum* (*Cm*) is a murine-specific species commonly employed to study roles of both host and chlamydial factors during infection in a small animal model (7, 10).

Genes encoded within the so called chromosomal plasticity zone (PZ) (11) have become an area of interest since species-specific PZ content could correlate with variation in tissue tropism, cellular infection biology, and pathogenesis manifested among *Chlamydia* spp. The hypervariable PZ ranges in size from ca. 5 kb in avian-specific *C. avium* to ca. 81 kb in *Cm* (2). Species variability in genes encoding proteins homologous to large clostridial cytotoxins (LCT) represents one noteworthy polymorphism within the PZ. The prototypical LCT, *Clostridium difficile* TcdB, is a glycosyltransferase (GT) that modifies and inactivates Rho and Ras GTPases to interfere with actin polymerization. Like other LCTs, TcdB glycosyltransferase activity requires a catalytic Asp-X-Asp (DXD) and UDP-Glucose-binding (W) motifs, while a cysteine protease triad (Cys-His-Asp) mediates self-cleavage of endocytosed toxin to release the GT domain into the cytosol (12). In *Cm*, the toxin (*tox*) locus encodes three homologous (>60% similarity) proteins containing DXD, W, and CHD motifs that are encoded by tandem genes designated *tc0437*, *tc0438*, and *tc0439* (11). In contrast, truncations or frame-shifts result in retention of coding sequence for a ca 72 kDa protein retaining only the DXD and W motifs in *Ctr* urogenital strains (13). Putative toxin genes are entirely missing from LGV serovars of *Ctr* (2).

The role of putative chlamydial cytotoxins remains elusive. Homology to TcdB suggests the modulation of actin polymerization. Indeed, the presence of complete or partial putative cytotoxins correlates with rapid (1–4 h) collapse of the host actin-based cytoskeleton during high multiplicity tissue culture infections with *Cm* and *Ctr* serovar D (13)—a phenomenon termed immediate toxicity (14). Immediate toxicity is not manifested during infections with *Ctr serovar* L2 which lacks toxin genes, leading to the hypothesis that these proteins exert species-specific effects on the actin cytoskeleton during chlamydial entry (15). Consistent with this notion, ectopic expression of *Ctr* D CT166 in HeLa cells results in cell rounding with a modest decrease in Rac1 abundance (16) and decreased immunodetection of Ras abundance with glycosylation-sensitive antibodies (17). These effects required the presumed DXD motif. However, mobilization of *tc0437–0439* to *Ctr* L2 via lateral gene transfer (LGT) did not confer a toxicity phenotype (18). Single gene inactivation of *Cm tc0437*, *tc0438,* or *tc0429* via chemical mutagenesis yielded somewhat ambiguous results with regard to cytotoxicity and did not significantly impact recovery of shed bacteria in an intravaginal murine infection model (19).

The development of genetic techniques in *Chlamydia* is providing a means to more conclusively address infection biology. Approaches include random chemical (20, 21), temperature-sensitive (22), or transposon-based (23–25) mutagenesis; targeted gene disruption via Group II intron insertion (26, 27); complete gene deletion via allelic exchange (28, 29); or conditional knockdown using CRISPRi (27) or small RNAs (30). Transformation and mutagenesis have become common in the model organisms *Ctr* L2 and *Cm* (31). Gene deletion studies have thus far focused on the removal of narrow DNA stretches containing single or adjacently positioned genes. Lateral gene transfer (LGT) can accomplish transfer of larger DNA elements when *Chlamydia* strains co-occupy a single inclusion and homologous recombination results in replacement of recipient DNA with that of a donor strain (32). Random mobilization of chromosome segments as large as 790 kb has been documented (33), and interspecies LGT has also been leveraged to interrogate gene function (34).

Given the potential for functional redundancy, we used fluorescence-reported allelic exchange mutagenesis (FRAEM) to entirely delete *tc0437–0439* in a single strain. Lateral gene transfer was used to restore the toxin locus in a complementing strain. In tissue culture, complete loss of the *tox* locus did not significantly decrease intracellular growth, immediate cytotoxicity, or adherence to host cells. Interestingly, the Δ*tox* strain displayed reduced invasion efficiency in tissue culture. During infection of mice, Δ*tox* was attenuated for shedding from the lower genital tract but not in maintaining upper genital tract infectious burden. Transcervical inoculation revealed a requirement of the toxins in manifesting both acute and chronic pathology in the oviduct. Collectively, our results are consistent with a function of putative cytotoxins in *Cm* during chlamydial entry and indicate a potential tissue-specific role of these proteins in *Chlamydia*-induced pathology during *in vivo* infection.

## RESULTS

**Fig 1:**
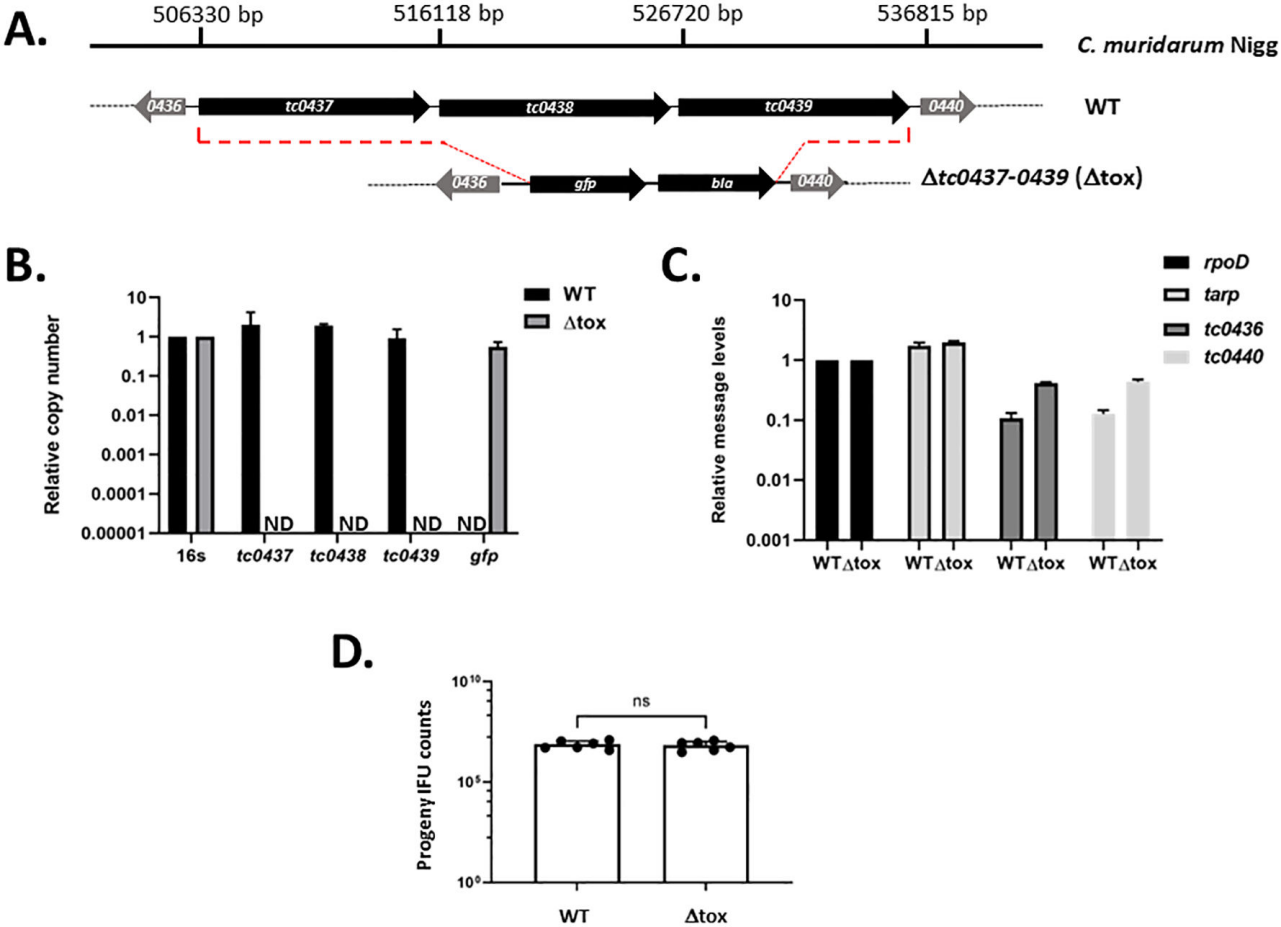
Deletion of the *tox* locus from *C. muridarum*. (**A**) Schematic of toxin genes *tc037–0439* in relation to the *Cm* (WT) genome and their replacement with a *blaM-gfp* cassette in Δ*tox*. Triplicate McCoy monolayers were equivalently infected with WT or mutant (Δ*tox*) and DNA or RNA was harvested from cultures at 24 h post infection. (**B**) The presence of chromosomal *tc0437–0439* and *gfp-*specific signal was evaluated in DNA samples via qPCR where values were normalized to 16 s content (ND, none detected). (**C**) Gene-specific mRNA levels for *tarp*, and toxin-flanking genes *tc0436* and *tc0440* were assessed during WT and Δ*tox* samples via qRT-PCR analysis of whole-culture RNA. Values were normalized to *rpoD* expression levels and no statistically significant differences were found. (**D**) McCoy cells were equivalently infected with WT or Δ*tox Cm* and cultures harvested at 24 h for enumeration of progeny EBs (IFU counts). All data are presented as average values with standard deviation from triplicate samples. Independent experiments were conducted three times and a representative experiments are shown. Progeny counts are presented as averages (bars) of triplicate samples counted in duplicate (closed circles). Statistical significance was evaluated by Student’s *t*-test (ns, not significant).

FRAEM-mediated gene deletion in *Chlamydia* has proven useful for inactivation of relatively small (ca. 1–2 kb) genetic elements. We wondered if the approach could also enable deletion of large segments of the chromosome and chose to target the chlamydial plasticity zone since this locus encodes species-specific genes having a lower likelihood of being essential. We targeted the cytotoxin (*tox*) locus containing three tandem genes (*tc0437*, *tc0438*, and *tc0439*) encoding highly similar proteins and spanning ca. 30 kb within the plasticity zone of *Cm*. FRAEM was used for complete gene deletion (start codon of *tc0437* to the stop codon of *tc0439*) and replacement of putative cytotoxin genes with a selection cassette encoding GFP and BlaM (Fig. 1A). To generate the Δ*tox* strain, DNA representing ~3  kb sequence flanking the *tc0437–tc0439* locus was amplified from *Cm*. These upstream and downstream 3 kb sequences were mobilized sequentially via Gibson Assembly into pKW-CM*ori* and positioned 5′ or 3′ of the *blaM-gfp* cassette, respectively. The resulting pKW-tox was transformed into *Cm* WT under PenG selection, and transformants were passaged to allow allelic replacement mutagenesis. PenG-resistant, GFP-positive inclusions were harvested, and loss of *tc0437–tc0439* was assessed in a clonal isolate via qPCR of extracted DNAs normalized for 16 s content. Successful deletion of *tc0437–tc0439* was indicated by detection of *gfp* but not *tc0437*, *tc0438*, and *tc0439* (Fig. 1B). Whole-genome sequencing confirmed the loss of the 30,234 bp segment encoding *tc0437–tc0439* in *Cm* Δ*tox*. Analysis of small-nucleotide polymorphisms (SNP) revealed WT and Δ*tox* genomes were otherwise identical (Table S1) with the exception of a frame-shift mutation in *tc0412* of the Δ*tox* strain.

To further validate the Δ*tox* strain, we examined levels of *tc0436* and *tc0440* mRNA to ensure that expression of these flanking genes was not disrupted. Whole culture RNA was extracted from *Cm* WT and Δ*tox* at 24 h post infection and analyzed via qRT-PCR (Fig. 1C). Message levels were normalized to *rpoD,* and we tested *tarp* levels as an off-target control. Similar to *tarp*, the message for both *tc0436* and *tc0440* was not decreased in the Δ*tox* strain, indicating that the transcription of these genes is not disrupted. Finally, we tested the intracellular fitness of Δ*tox* in comparison to the parent WT strain (Fig. 1D). McCoy cell monolayers were equally infected with *Cm* WT or Δ*tox*. Cultures were harvested at 24 h and passaged onto fresh monolayers for enumeration of progeny EBs. Progeny IFU counts were similar for WT and Δ*tox*, indicating that loss of *tc0437–tc0438* does not overtly impact intracellular growth in tissue culture.

**Fig 2:**
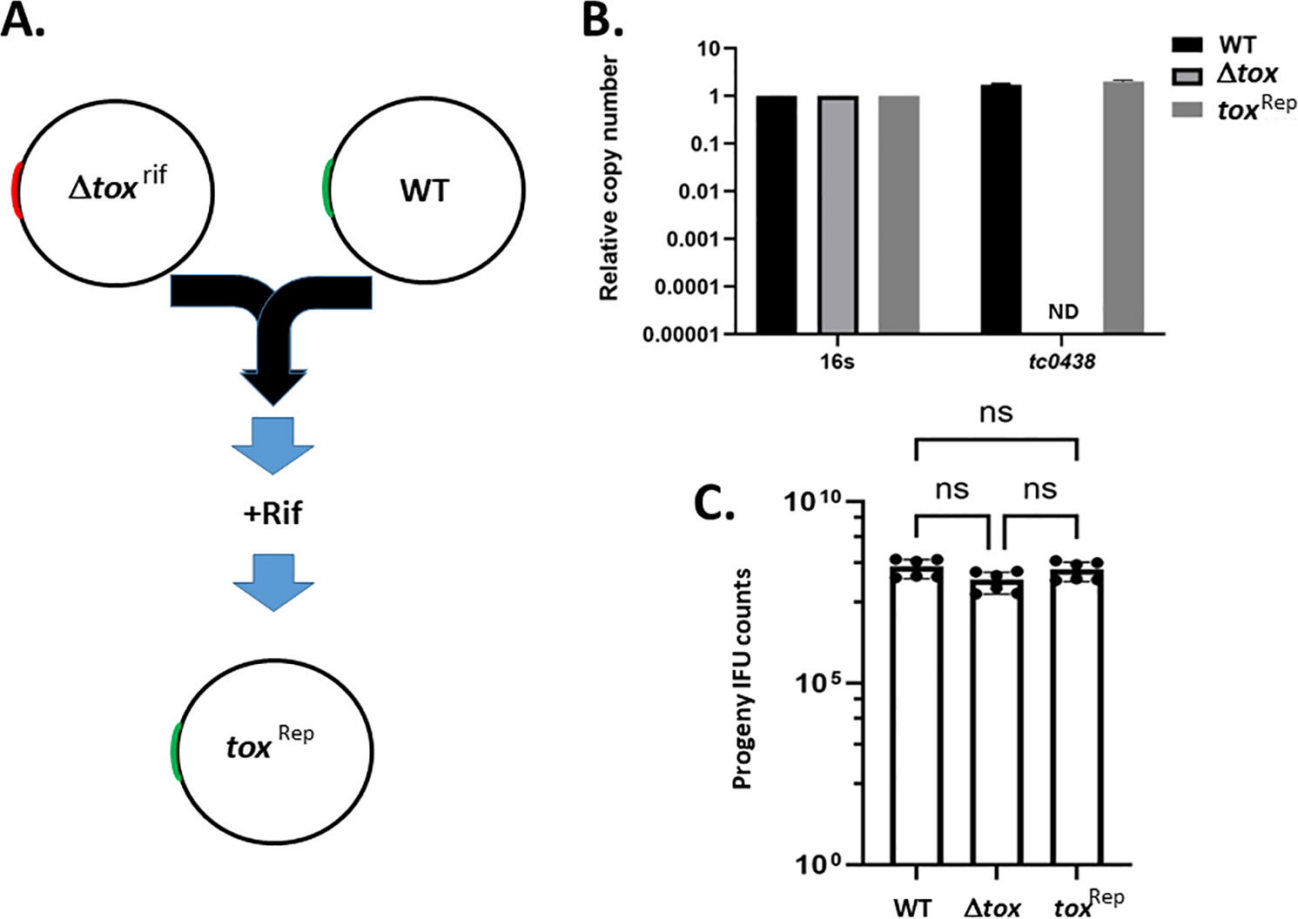
Generation of *tox* locus repaired strain for complementation studies. (**A**) Schematic for restoration of the *tox* genes to the Δ*tox* strain. A rifampin (rif) resistant isolate of Δ*tox* lacking the *tox* locus (red) was co-cultured with rif-sensitive WT *Cm* where *tox* genes (green) are intact to allow lateral gene transfer. A recombinant strain where the *tox* deletion was repaired (*tox*^Rep^) by transfer of genes from WT into the Δ*tox* chromosome was isolated by sequential culture with 15 ng rifampin and harvest of rif-resistant, non-fluorescent inclusions. Whole-culture material was harvested from McCoy cells equivalently infected with WT, mutant (tox) or repaired (tox^Rep^) strains for 24 h. (**B**) DNA was probed with gene-specific primers via qPCR to establish relative abundance of *tc0438*. Strain-specific values were normalized to 16 s content (ND, none detected). (**C**) McCoy cells were equivalently infected with indicated strains and cultures harvested at 24 h for enumeration of progeny EBs (IFU counts). All quantitative data are presented as average values with standard deviation from triplicate samples of a representative (of three total) experiment. Progeny counts are presented as averages (bars) of triplicate samples counted in duplicate (closed circles). Statistical significance was evaluated by one-way ANOVA with multiple comparisons (ns, not significant).

We next sought to generate a strain in which the *tox* locus was restored for ensuing phenotypic studies, but the *tc0437–tc0439* locus deletion is too large to generate shuttle vectors. We elected to leverage lateral gene transfer to overcome this barrier since studies have shown that large segments of the chlamydial chromosome can be transferred (Fig. 2A). The Δ*tox* strain was first subjected to cultivation in the presence of increasing rifampin concentrations to select Rif-resistant chlamydiae to enable a selection for the mutant-derived chromosome. A clonal strain was derived by limiting dilution and subsequently co-cultured with *Cm* WT. Rifampin selection was applied after multiple co-infection passages, and material was used to infect McCoy cells in a 384-well plate. These wells were screened for Rif-resistant inclusions that lacked GFP signal. A clonal strain was isolated by subsequent limiting dilution and tested for the presence of *tc0438* as an indicator for repair (Rep) of the *tox* locus (Fig. 2B). Whole culture DNA was extracted from 24 h cultures of WT, Δ*tox*, and *tox*^Rep^ and probed via qPCR. Values were normalized to 16 s. We detected a signal for *tc0438* in both WT and *tox*^Rep^ DNA, indicating restoration of the *tox* locus. Whole-genome sequencing of genomic DNA confirmed restoration of the entire tox locus in the *tox*^Rep^ strain. Finally, we performed an additional progeny assay to test for any fitness alterations. Cultures were harvested at 24 h and passaged onto fresh monolayers for enumeration of progeny EBs (Fig. 2C). Progeny IFU counts were similar for three strains, indicating no difference in intracellular growth.

**Fig 3:**
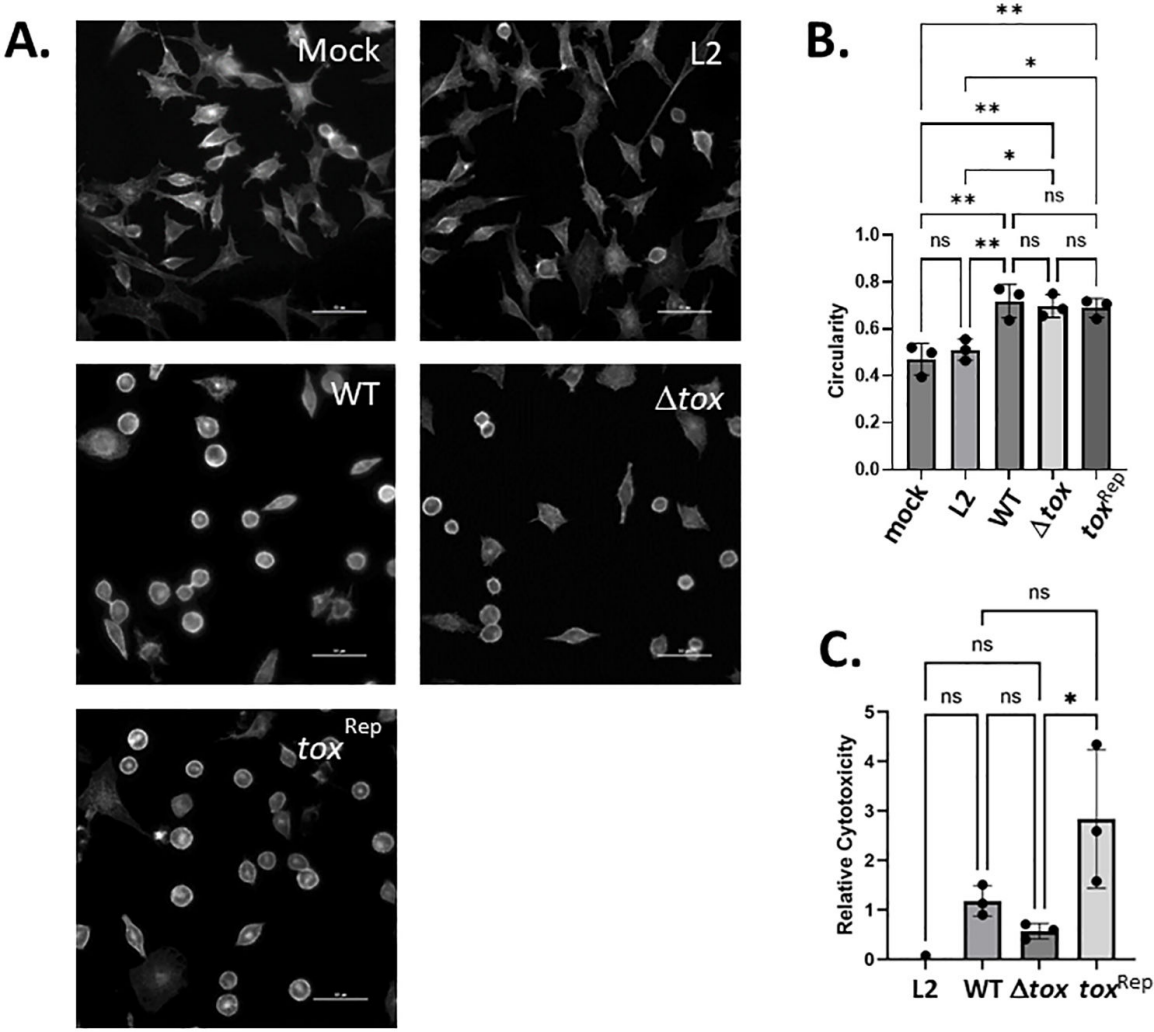
Absence of *tox* genes does not correlate with loss of immediate toxicity. McCoy monolayers were mock-treated or infected at an MOI of 500 with *Ctr* L2, WT *Cm*, or *Cm* where toxin genes were lacking (Δ*tox*) or restored (*tox*^Rep^). (**A**) Representative immunofluorescence images are shown from 4 h cultures stained with phalloidin for visualization. (**B**) Cell rounding was used as an indicator of immediate toxicity and was quantified by measuring circularity (4pi(area/perimeter^2^)) of cells. (**C**) Overt cellular toxicity was assessed via quantification of LDH release by infected monolayers. McCoy cells were equally infected for 4 h with *Ctr* L2 or *Cm* WT, Δ*tox*, or *tox*^Rep^ Cm and LDH levels were measured in cell-free culture supernatants. Relative cytotoxicity was calculated by comparing spontaneous LDH release in mock-infected cells to infection-induced LDH release. All quantitative data are presented as average (bars) values with standard deviation from triplicate samples (closed circles) of a representative experiment. Cell rounding experiments were conducted three times, whereas four independent LDH assays were conducted. Statistical significance was evaluated by one-way ANOVA with multiple comparisons (ns, not significant; *, *P* < 0.01, **, *P* < 0.001).

To explore the potential cytotoxic activity of putative cytotoxins, we tested criteria ascribed to immediate cytotoxicity including cell rounding and release of lactate dehydrogenase (LDH). McCoy cells were mock-treated or equivalently infected with *Ctr* L2, *Cm* WT, Δ*tox*, or *tox*^Rep^ at an MOI of 500. At 4 h post infection, cultures were fixed and phalloidin stained to visualize cell morphology (Fig. 3A) and calculate circularity of cells (Fig. 3B). As expected, McCoy cells infected with L2 exhibited a phenotype similar to mock-infected cells with limited cell rounding. Consistent with previous observations (15), cells infected with *Cm* WT exhibited a significant (*P* = 0.0035) increase in cell rounding compared to mock infected cells. Interestingly, neither Δ*tox* nor *tox*^Rep^ infected cells exhibited significant differences in (*P* = 0.47 and *P* = 0.83, respectively) cell rounding compared to cells infected with *Cm* WT indicating that the three putative cytotoxins do not have a role in manifesting these parameters of immediate cytotoxicity. We confirmed cell rounding in human epithelial HeLa cells (Fig. S2) since this line has been used in past studies (15, 19). We also examined LDH release into culture supernatants to test cell membrane integrity (Fig. 3C). Neither WT nor Δ*tox* stimulated LDH release above that observed for *C. trachomatis* L2, and Δ*tox* did not differ significantly from WT. We did note that LDH release elicited by the *tox*^Rep^ strain was highly variable, leading to a group average that was statistically different (*P* = 0.0432) from Δ*tox* but not WT. This was reproducible and could be due to consistent variability observed among *tox*^Rep^ replicates.

**Fig 4:**
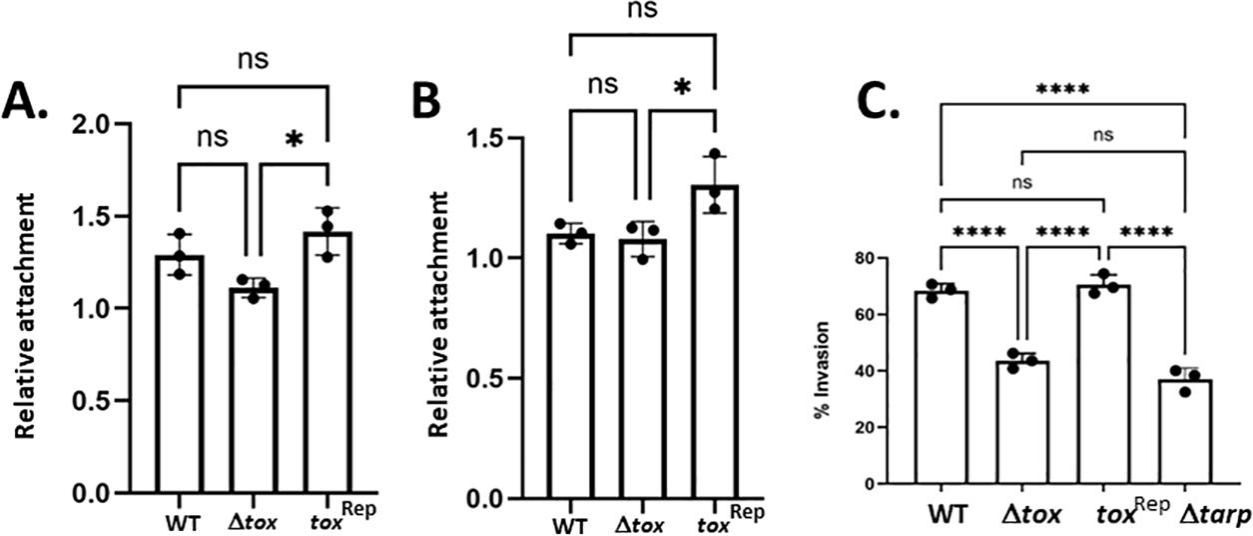
Loss of the *tox* locus does not impact *Cm* adherence to McCoy cells but does decrease invasion efficiency. Adherence of each strain to the host cell was evaluated by infecting McCoy cell monolayers untreated (**A**) or pretreated (**B**) with DEAE dextran. Two hours post infection, monolayers were thoroughly washed with HBSS, and the attached bacteria were quantified by assessing chromosome copy number via qPCR using 16s-specific primers. Data are presented as inoculum-normalized genome equivalents to indicate relative attachment. (**C**) Invasion was assessed by infecting McCoy cells at an MOI of 10 with *Cm* WT, Δ*tox*, *tox*^Rep^, or Δ*tarp* as a positive control. Cultures were paraformaldehyde fixed after 30 min and processed for inside-out staining to assess invasion efficiency. Data are represented as percentage of internalized chlamydiae. All data are represented as means (bars) with standard deviations from triplicate samples (closed circles) from representative experiments (of three total). Statistical significance was computed using one-way ANOVA with multiple comparisons (ns, not significant; *, *P* < 0.04; ****, *P* < 0.0001).

We next investigated whether loss of *tox* genes would affect adherence or invasion. Adherence was evaluated in McCoy monolayers that were either untreated or treated with DEAE-dextran for 15 min prior to infection to artificially increase attachment efficiency (35). Cells were infected with *Cm* WT, Δ*tox*, or *tox*^Rep^ at an MOI of 5. Monolayers were washed 3× with HBSS at 2 h post infection, and cell-associated bacteria were quantified via qPCR-based genome quantification from isolated whole-culture DNA. As expected, dextran treatment increased abundance of cell-associated chlamydiae (Fig. S1). However, there was no significant difference in the level of overall attachment comparing WT and Δ*tox* or *tox*^rep^ in either condition (Fig. 4A and B). Although modest, significant differences were detected when comparing *tox*^rep^ with Δ*tox* (*P* = 0.0247 and 0.0379 for – and +dextran, respectively). It is unclear, however, whether these differences are physiologically relevant. We next assessed the role of putative cytotoxins in invasion by infecting McCoy cells with *Cm* WT, Δ*tox*, or *tox*^Rep^ at an MOI of 10. Monolayers were also similarly infected with *Cm* Δ*tarp* as a positive control. Cells were fixed after 30 min at 37°C, processed for inside-out staining, and the percentage of internalized EBs calculated for each strain. As previously shown (36), *Cm* Δ*tarp* exhibited decreased invasion efficiency. The absence of putative cytotoxins in Δ*tox* also led to a significant decrease in invasion efficiency compared to WT. Furthermore, the level of invasion evident for Δ*tox* was comparable to that of the Δ*tarp* strain (*P* < 0.001 for both). In aggregate, these data indicate that Tox proteins are required for efficient invasion but not initial attachment to cells.

**Fig 5:**
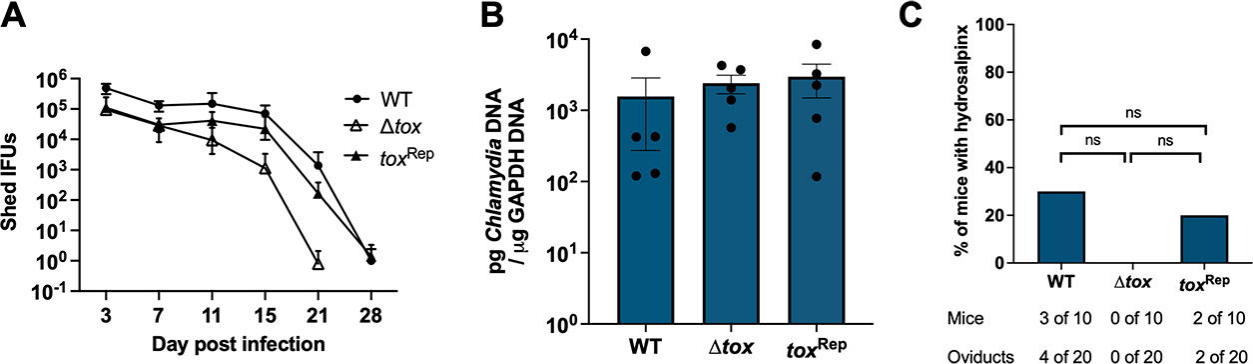
Deletion of *tox* locus causes mild defect in vaginal shedding yet similar levels of ascension and hydrosalpinx after intravaginal infection. C57Bl/6 female mice were infected intravaginally with 10^5^ IFU of *C. muridarum* WT, Δ*tox,* or *tox*^Rep^. (**A**) IFU enumerated from vaginal swabs collected at days 3–28 post infection. Data presented as means +/− standard deviation from five mice/group/time point. Statistical significance was assessed by two-way RM ANOVA followed by Tukey’s multiple comparisons test; *P* < 0.05 for WT vs other strains at day 3 and 7. (**B**) Ascension at day 7 post intravaginal infection as measured by qPCR on upper genital tract homogenates. Statistical significance was assessed by one-way ANOVA followed by Tukey’s multiple comparisons test and found to be not significant. (**C**) Frequency of hydrosalpinx at 51 days post intravaginal infection, pooled from two independent experiments. Statistical significance was assessed by Fisher’s exact test; ns, not significant.

We next sought to explore the contributions of the *tox* locus to pathogenicity in the female genital tract. Established mouse models of intravaginal and transcervical infection were used to measure bacterial burden and pathology at acute and chronic times post infection. C57Bl/6 female mice were infected intravaginally with 10^5^ IFU of *Cm* WT, Δ*tox*, or *tox*^Rep^, and vaginal shedding was measured from day 3 to 28 post infection. *Cm* Δ*tox* exhibited a minor defect in shedding and was cleared from the lower genital tract one week before *Cm* WT or *tox*^Rep^ (Fig. 5A). By contrast, all *Cm* strains were equally able to ascend to the upper genital tract resulting in similarly high bacterial burden as measured by qPCR on upper genital tract homogenates at days 7 and 16 post-intravaginal infection (Fig. 5B; Fig. S3A). When upper genital tract gross pathology was assessed at 51 days post intravaginal infection, the frequency of hydrosalpinx was found to be low and did not differ significantly between groups (Fig. 5C; Fig. S3B).

**Fig 6:**
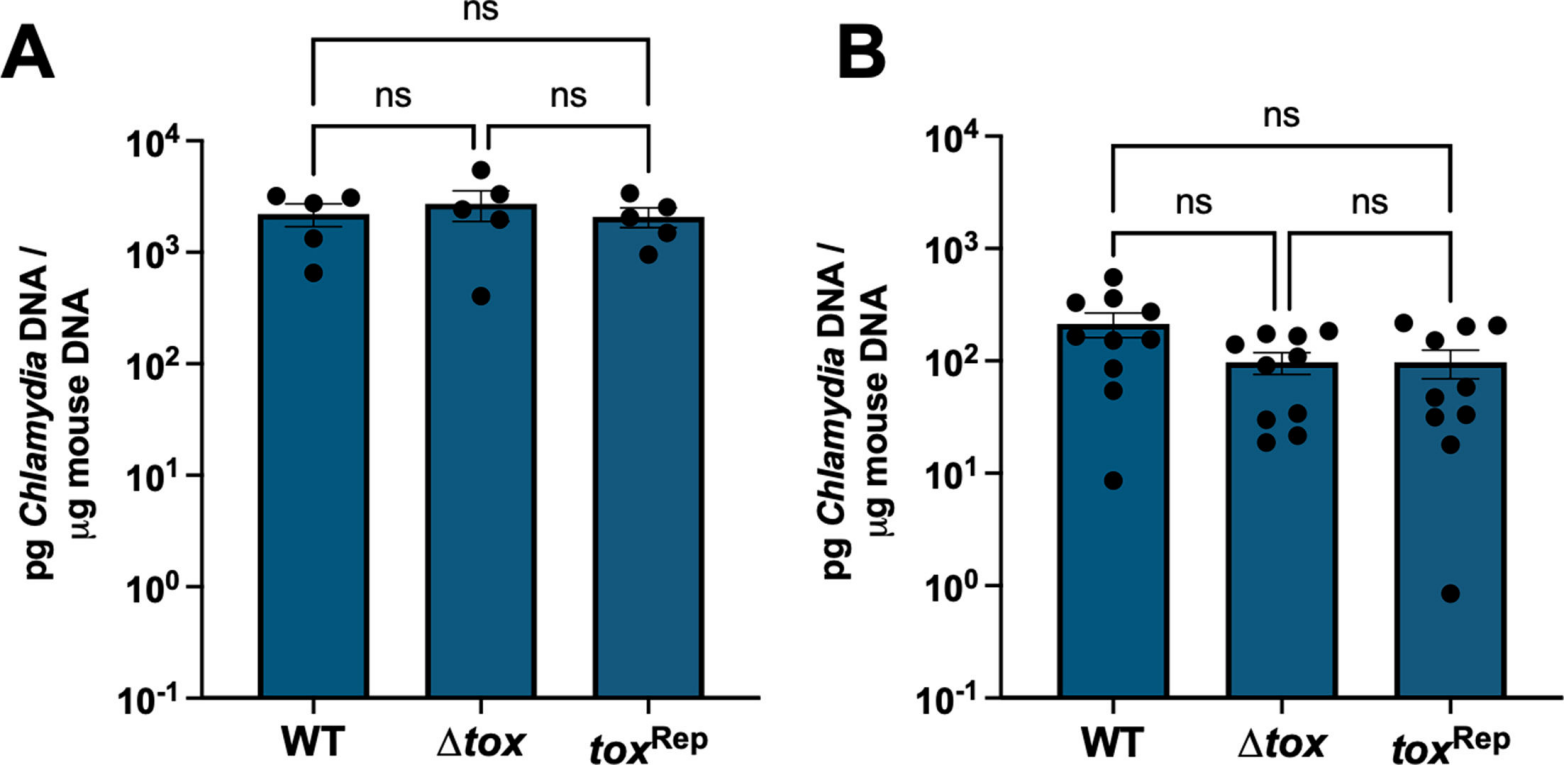
Deletion of *tox* locus does not impact bacterial burden in the upper genital tract after transcervical infection. C57Bl/6 female mice were infected transcervically with 10^6^ IFU of *C. muridarum* WT, Δ*tox,* or *tox*^Rep^. At 3 days (**A**) and 7 days (**B**) post-infection, bacterial burden in the upper genital tract was quantified by qPCR. Data are plotted as mean +/− SEM. Data in B are pooled from two independent experiments. Statistical significance was assessed by one-way ANOVA followed by Tukey’s multiple comparisons test; ns, not significant.

**Fig 7:**
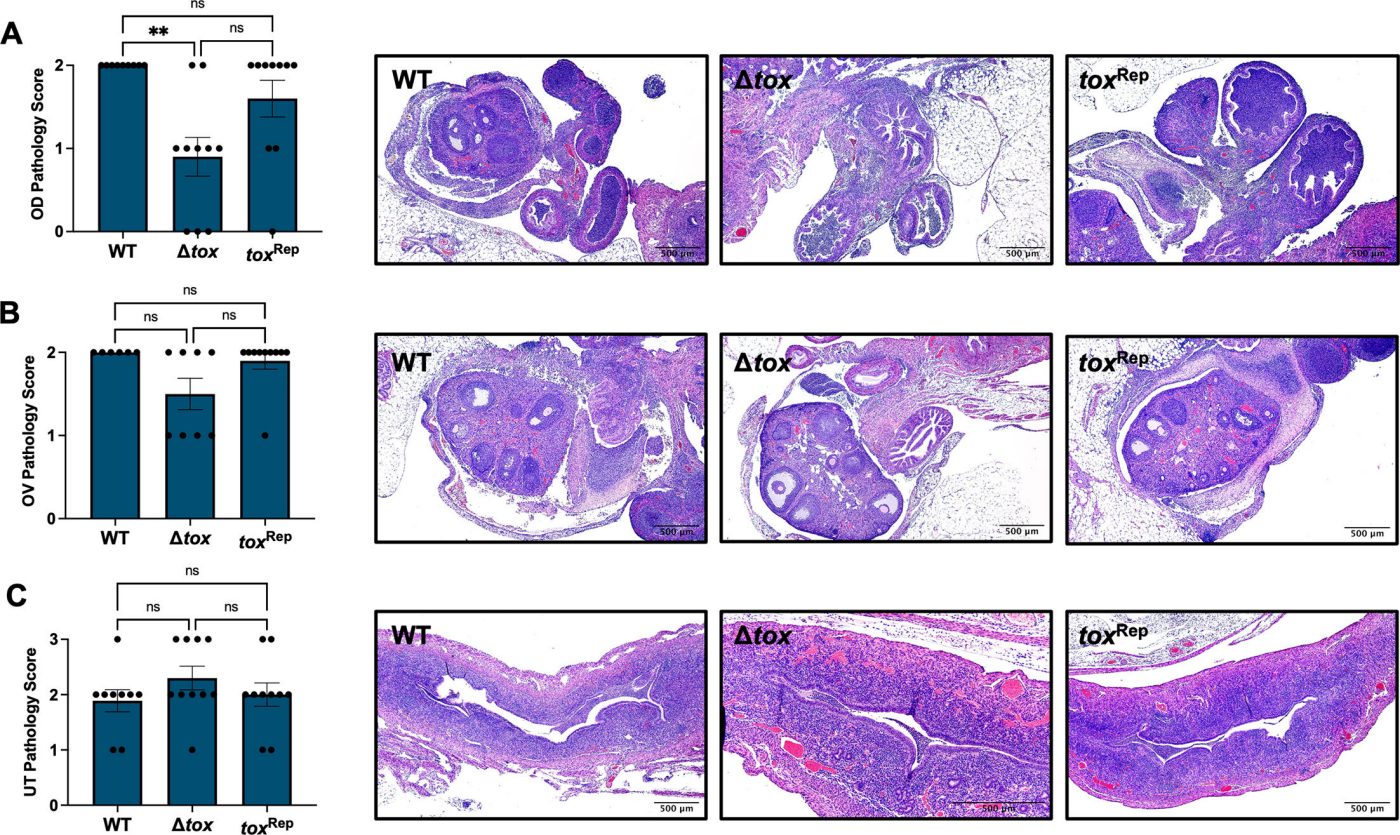
Acute oviduct pathology is ameliorated by the deletion of *tox* locus. Seven days after transcervical infection of C57Bl/6 mice with 10^6^ IFU of *C. muridarum* WT, Δ*tox,* or *tox*^Rep^, female genital tracts were excised and histopathology was assessed on randomized, anonymized slides. (**A–C**) Pathology scores and representative images of H&E stained oviduct (OD, **A**), ovary (OV, **B**), and uterus (UT, **C**). Statistical significance was assessed by Kruskal-Wallis test followed by Dunn’s multiple comparisons test; **, *P* = 0.0017; ns, not significant.

Therefore, we turned to the transcervical route of inoculation to model upper genital tract infection and pathology. C57Bl/6 female mice were infected transcervically with 10^6^ IFU of *Cm* WT, Δ*tox*, or *tox*^Rep^, and bacterial burden in UGT homogenates was measured by qPCR. *Cm* WT, Δ*tox*, and *tox*^Rep^ were equally capable of infecting the upper genital tract at day 3 (Fig. 6A) and day 7 (Fig. 6B), consistent with the equivalent burdens seen in the UGT after intravaginal infection (Fig. 5B). By contrast, the deletion of the *tox* locus did result in changes to immunopathology phenotypes. Seven days after transcervical infection with *Cm* WT, Δ*tox*, or *tox*^Rep^, histopathology was assessed on anonymized H&E-stained uterus, ovary, and oviduct. Robust immunopathology, largely characterized by dense cellular infiltration, was present in the oviducts of mice infected with *Cm* WT and *tox*^Rep^ yet was significantly reduced in mice infected with *Cm* Δ*tox* (Fig. 7A, *P* = 0.0017). A similar trend toward reduction in cellular infiltration was observed between the ovary and bursa after *Cm* Δ*tox* infection compared to controls, whereas thickening of the ovarian bursa itself was observed after infection with all three strains, and ovary pathology scores were not significantly different (Fig. 7B). Immunopathology in the uterus was moderate and consistent between *Cm* WT, Δ*tox*, and *tox*^Rep^ (Fig. 7C), suggesting that the *tox* locus may contribute specifically to oviduct but not uterine histopathology.

**Fig 8:**
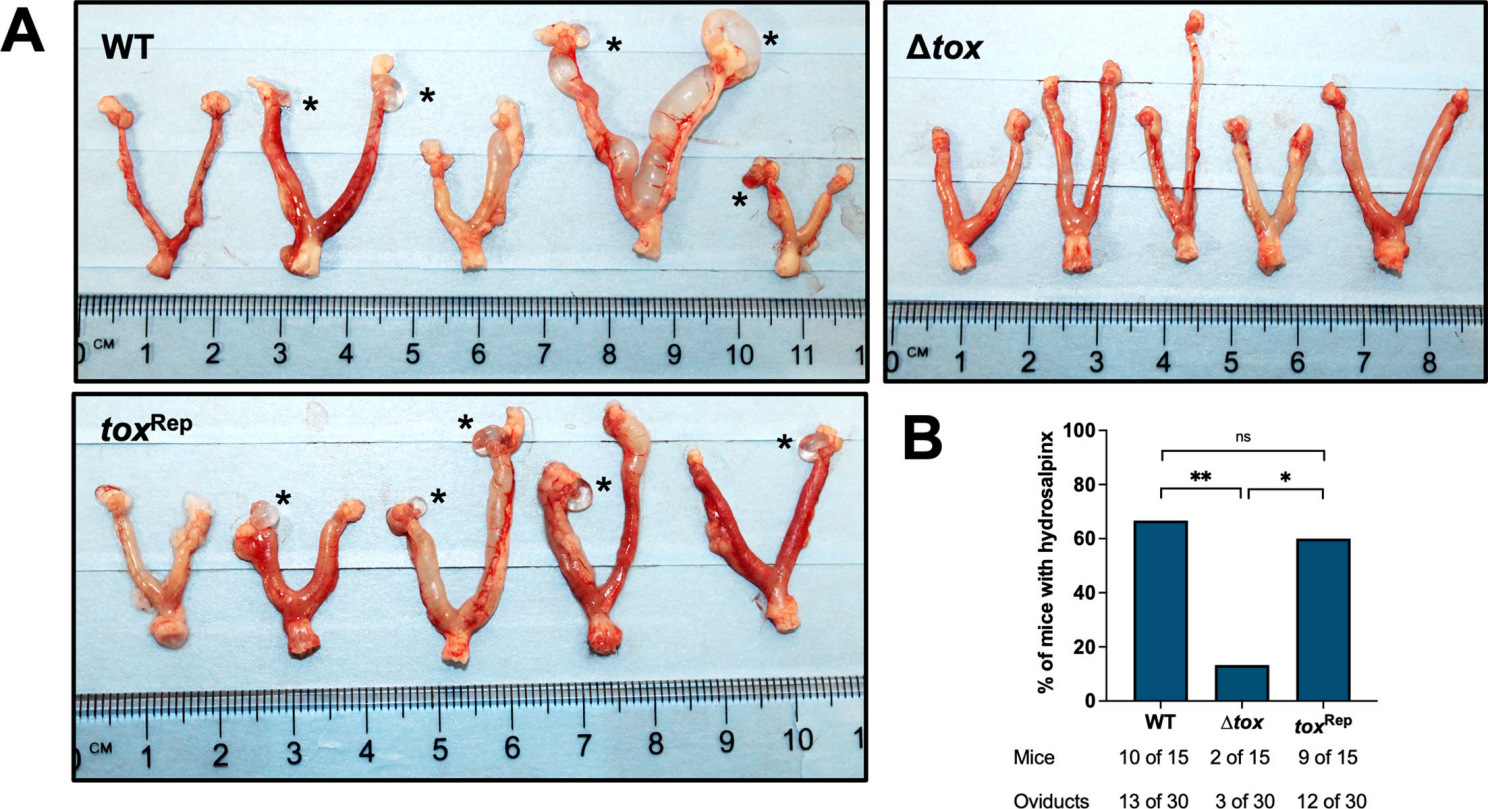
Frequency of hydrosalpinx after transcervical infection is reduced by deletion of *tox* locus. Fifty-one days after transcervical infection of C57Bl/6 mice with 10^6^ IFU of *C. muridarum* WT, Δ*tox,* or *tox*^Rep^, female genital tracts were assessed for gross pathology. (**A**) Representative images of excised upper genital tracts; asterisk indicates hydrosalpinx. (**B**) Frequency of hydrosalpinx pooled from two independent experiments. Statistical significance was assessed by Fisher’s exact test; **, *P* = 0.0078, *, *P* = 0.0209, ns, not significant.

**Fig 9:**
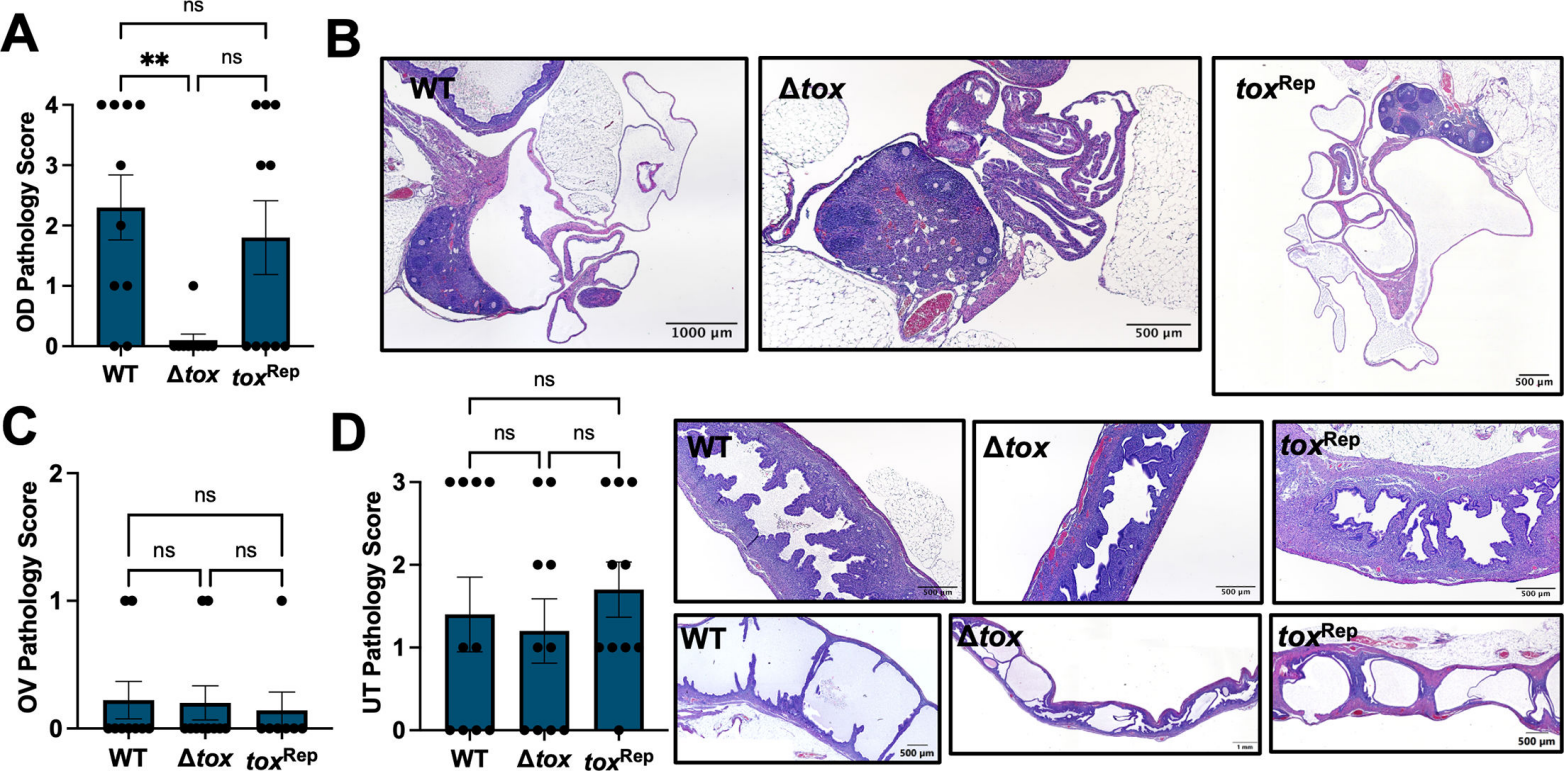
Chronic oviduct pathology is ameliorated by deletion of *tox* locus. Fifty-one days after transcervical infection of C57Bl/6 mice with 10^6^ IFU of *C. muridarum* WT, Δ*tox,* or *tox*^Rep^, female genital tracts were excised and histopathology was assessed on randomized, anonymized slides. (**A**) Oviduct (OD) pathology scores. (**B**) Representative images of H&E-stained oviduct and ovary. (**C**) Ovary (OV) pathology scores. (**D**) Pathology scores and representative images of H&E-stained uterus (UT). Two images of the uterus are shown from each infection condition to represent the within-group variability of horn dilation that was present after infection with all three bacterial strains. Statistical significance was assessed by Kruskal-Wallis test followed by Dunn’s multiple comparisons test; **, *P* = 0.0077; ns, not significant. Data are representative of two independent experiments.

As with acute infection, the deletion of the *tox* locus also improved oviduct pathology during chronic infection. Gross pathology (Fig. 8) and histopathology phenotypes (Fig. 9) were assessed at day 51 after transcervical infection with *Cm* WT, Δ*tox*, or *tox*^Rep^. The frequency of hydrosalpinx was significantly reduced to 13% in *Cm* Δ*tox*-infected mice compared with ~60% in *Cm* WT and *tox*^Rep^ -infected controls (Fig. 8B). Significant differences were also observed at the histopathological level specifically in the oviduct, where *Cm* WT and *tox*^Rep^ induced severe pathology characterized by luminal edema that was not observed after *Cm* Δ*tox* infection (Fig. 9A and B). Immunopathology in the ovary was uncommon and, when present, mild across all infection conditions (Fig. 9B and C), except for the distortion of ovary morphology caused by adjacent hydrosalpinx (Fig. 9B, WT and *tox*^Rep^). As with chronic oviduct pathology, chronic uterine pathology was characterized by luminal edema rather than the cellular infiltration that was observed at day 7 post infection (Fig. 9D). The severity of uterine pathology at this timepoint was highly variable, ranging from some horns with no pathology and others with confluent dilation (Fig. 9D). Variable uterine pathology phenotypes were found equivalently after infection with *Cm* WT, Δ*tox*, or *tox*^Rep^ (Fig. 9D), again suggesting that the contributions of the *tox* locus to pathology may be localized to the oviduct rather than the uterus. Bacterial burden in the upper genital tract at this time point was not detectable by qPCR for any strain (data not shown).

## DISCUSSION

The *tox*-containing chromosomal region of the *Cm* PZ represented an ideal deletion target for several reasons. First, chemically mutagenized studies harboring individually inactivated *tox* genes remained viable (19), decreasing the possibility of the locus being essential for growth. The *tox* genes *tc0437–tc0439* each encode proteins of ca. 360 kDa, and TC0437 shows 61% and 63% similarity to TC0438 and TC0439, respectively. Chemically induced single mutant strains were not able to address the potential for functional redundancy among these homologous proteins. Apparent hypervariability of encoded proteins also draws interest regarding species-specific infection biology. Animal species *C. suis* and *C. pecorum* each express two *tox* genes, *C. caviae* and *C. psittaci* encode a single gene, and *C. abortus* lacks a *tox* gene altogether (2). Among human species, *Ctr* serovar D–G, I, and K express a single, truncated protein of ca. 72 kDa containing only domains necessary for GT activity. Serovars H and J contain full-length DNA, but an apparent nonsense mutation yields a truncated protein similar to serovar D. Serovars A–C are further truncated, and it is unclear whether a protein is expressed from this locus. A corresponding gene is completely absent from *Ctr* L1-3 and *C. pneumoniae* (2, 13). Finally, previous comparative genomics, predicted GT function similar to LCTs, and ectopic expression approaches suggested a role of *tox* genes in species-specific immediate host cell cytotoxicity during chlamydial entry (15–17). In the absence of tractable genetics, however, a more definitive characterization was not possible.

The advent of reproducible transformation in *Chlamydia* has ushered in a new era of genetic malleability that includes allelic exchange mutagenesis for complete removal of coding sequences (31, 37). Two techniques have been generated leveraging either a non-replicating shuttle vector expressing lambda red recombinase (28) or a conditionally replicating plasmid relying on endogenous recombination machinery (29). We extended the utility of FRAEM herein to target large stretches of DNA. The deletion of the ca. 30 kb *tox* locus from the *C. muridarum* chromosome was accomplished along with repair of the lesion using LGT. Characterization of mutant infectivity in tissue culture and the murine infection models revealed surprising results including the absence of mutant impact on immediate host cell toxicity. Instead, loss of the *tox* proteins resulted in decreased host cell invasion and attenuated pathology in the oviduct of infected mice. Overall, our data underscore the importance of these proteins in *Cm* infection and provide an example of how advances in chlamydial genetics are providing a more definitive understanding of infection biology.

The term “immediate toxicity” was originally coined to describe cell rounding that occurred within hours of high MOI infection of *C. psittaci* on L929 murine fibroblasts that was independent of *de novo* protein synthesis (38). TC0437–0439 were originally designated putative cytotoxins based on the presence of confined domains similar to active-sites of a large exotoxin superfamily produced by bacterial pathogens that includes prototypical TcdA and TcdB of *Clostridium* (15). The presence of complete or partial chlamydial *tox* genes also correlated with species-specific immediate toxicity, reinforcing the cytotoxin designation (13, 15). For example, Belland et al. (15) showed cell rounding and collapse of the actin cytoskeleton during high MOI infections of HeLa cells with either *C. muridarum* or *C. trachomatis* serovar D. TcdA/TcdB bind UDP-glucose and glucosylate Rho family GTPases to inhibit GTP binding and therefore interfere with actin polymerization (12). Although chlamydial Tox proteins contain conserved domains necessary for UDP-glucose binding and glycosyltransferase activity, direct functional evidence is lacking (12). Ectopic expression studies using *Ctr* serovar D CT166 are consistent with GT activity. Notably, CT166 expression causes rounding of HeLa cells and can be overcome by the co-expression of constitutively active Rac1 or the deletion of the CT166 DXD residues (16). Expression of WT, but not DXD-deficient, CT166 also results in decreased immunodetection of Ras GTPase with glucosylation-sensitive antibodies (17). TC0437–0439 are *de novo* synthesized within hours of infection (19), but immunoblot analysis indicates a pool of EB-localized toxin in both *Cm* and *Ctr* serovar D (15). Based on expression and potential contributions to immediate toxicity, chlamydial toxins were predicted to function as early as during the entry process.

McCoy murine fibroblasts still exhibited cell rounding when infected with *Cm* Δ*tox* and overt lysis (LDH release) did not differ from WT. Loss of *tox* genes also failed to impact cell rounding during infection of HeLa cells (Fig. S2) as described by Belland et al. (15). We conclude TC0437–0439 are not required for immediate toxicity and are consistent with observations that inter-species transfer of the *Cm tox* genes to *Ctr* L2 did not confer an immediate cytotoxicity phenotype (18). These data do not rule out a functional role of Tox proteins in modulating actin polymerization but do indicate that changes to the working model of Tox function are warranted. Regardless, the putative toxins are dispensable for *in vitro* intracellular development. Consistent with single mutant strains (19), progeny from Δ*tox* were comparable to WT at 24 h post infection.

Our WT parent *C. muridarum* closely resembled that used in immediate toxicity studies reported by Rajaram et al. (19). SNPs were largely conserved with the exception that our WT strain lacked polymorphisms in coding sequences for 23S rRNA at position 967487 and had differing nucleotides in *tc0437* and *tc0324* at position 515,204 and 404884, respectively. One caveat to our studies is that both our Δ*tox* and *tox*^rep^ strains acquired a frame-shift mutation in *tc0412* during passaging and therefore also differ from the parent WT strain at this locus. A nucleotide at position 469 (of 1098) was deleted leading to a predicted truncated protein of 156 (of 366) residues. Passage-dependent inactivation of *tc0412* has been noted before (19), and mutations of *tc0412* in other backgrounds did not impact infection (19, 39). TC0412 is homologous (ca. 65% identity) to the inclusion membrane protein GarD (CT135) of *C. trachomatis*. GarD is also sensitive to inactivation during *in vitro* passaging (40) and has been associated with countering IFNγ-dependent, cell-intrinsic mechanisms (41–43). However, *C. trachomatis* and *C. muridarum* respond to IFNγ distinctly (44), raising the possibility of alternate functions for TC0412. The truncation of *tc0412* likely did not alter the interpretations from either our tissue culture or murine studies. The *tox*^rep^ strain was isogenic to Δ*tox* outside of the toxin locus and was isogenic to WT beyond the *tc0412* mutation and rif-resistant *rpoB* allele. This strain served as a control during infections and did not differ phenotypically from WT in progeny, cell rounding, invasion, and murine studies. Interpretation of LDH (Fig. 3C) and adherence (Fig. 4A and B) assays could be an exception. LDH release elicited by *tox*^rep^ was similar to WT but significantly higher and more variable when compared to Δ*tox*. Likewise, neither Δ*tox* nor *tox*^rep^ differed from WT in adherence assays, but adherence was significantly higher for *tox*^rep^ when compared to Δ*tox*. These small (ca. 1.2- to 3-fold) differences may not be physiologically relevant or could indicate a functional link between the toxins and TC0412. We cannot rule out the possibility that the combined loss of both TC0412 and toxins has a synergistic negative effect on LDH release and adherence.

Based on the presumed inhibitory effect of LCTs on actin polymerization, we were surprised to detect decreased invasion efficiency manifested by Δ*tox*. Rac1-mediated actin polymerization is important for chlamydial invasion (45). In addition, the type III secreted effector TarP cooperates with other effectors to promote Arp2/3-dependent actin polymerization to accomplish entry (46–48). Loss of *tc0437–0439* decreased invasion efficiency in magnitude similar to that of *Cm* Δ*tarp*. Actin polymerization during chlamydial infection is transient (49, 50), and invasion requires actin depolymerization (51). One possibility is that Tox proteins may contribute to entry by reversing actin polymerization. However, our data do not address whether potential GT activity or prototypical LCT function plays a role in invasion. Future experiments will be required to directly test the requirement of GT active site residues during invasion. Regardless of mechanism, contributions of Tox proteins in invasion may confer a degree of infectivity specificity since species or serovars exist that lack *tox* genes altogether.

As noted by Carlson et al. (13), the *Cm* toxins are most similar to designated lymphocyte inhibitory factor A (LifA) of pathogenic *E. coli*. For example, TC0438 exhibits 33.6% similarity by BLASTP to LifA representing the entirety of each protein. LifA suppresses T cell activation (52) and contributes directly to attachment and effacing lesions in epithelial cells (53). A LifA homolog from *C. pecorum* inhibited bovine T cell proliferation *in vitro* (54) and whether *Cm* Tox proteins share the T cell inhibitory potential remains to be determined. In enterohemorrhagic (55) and enteropathogenic (56) *E. coli*, the protein has been termed EHEC factor for adherence (Efa1) based on a requirement for attachment to epithelial cells. The expression of CT166 in HeLa cells modestly decreased serovar D infection (16), but these data represent artificial, ectopic over-expression and did not directly assay attachment. We did not observe a Tox-dependent difference in *Cm* adherence but cannot exclude an adhesin function due to likely redundant mechanisms such as the noted role of polymorphic outer membrane proteins in chlamydial attachment (57). It should also be noted that these studies employed the model fibroblast cell line McCoy and may not reflect requirements in columnar epithelial cells.

The *tox* locus has long been speculated to play a role in *Chlamydia* pathogenicity *in vivo* (13, 15). Not only do *tox* orthologs act as virulence factors in other pathogens, but within *Chlamydia* spp., the presence of *tox* genes correlates with the mucosal pathogenicity specific to the *Ctr* serovars D–K and *Cm*. To determine if this correlation is causative, we deleted *tox* from *Cm* and observed a reduction in immunopathology, a finding consistent with the hypothesis that Tox proteins act as virulence factors *in vivo*. The ameliorative effect on pathology was specific to the oviducts since immunopathology in the uterus was unchanged by *tox* deletion. Differences in pathology phenotypes were present as early as 7 days post infection and persisted beyond when bacteria are cleared from the tissues, implying that early events between virulence factors and innate immune signaling may drive chronic immunopathology outcomes. Oviduct-specific pathology after *Cm* infection has been shown to be dependent on IL-1α, with knockout mice having lower rates of hydrosalpinx (58). This effect is independent of bacterial burden, which was comparable in WT and IL-1α KO mice. TLR2 (59), MyD88 (60), and caspase-11 (61) have also been shown to play a role in driving chronic oviduct pathology without impacting *Cm* levels. After *Ctr* D transcervical infection, neutrophil depletion ameliorated upper genital tract pathology without reducing upper genital tract burden (62). Our studies also found no difference in burden in the UGT (uterus, ovary, and oviduct) but did not rule out the possibility that oviduct-specific burden may differ between strains and, if so, contribute to the observed differences in oviduct pathology. Future studies that determine the extent to which *Cm* Δ*tox* is impaired in infecting the oviduct specifically and/or activating innate factors like IL-1α, caspase 11, and neutrophils are warranted.

Tissue tropism is a hallmark of infection with *Chlamydia* spp., the mechanism for which remains an open area of investigation. Our studies did not address colonization of the gastrointestinal (GI) tract. *Cm* genital infections spread hematogenously to the GI, and the GI tract likely represents a natural reservoir for long-term colonization in mice (63). Indeed, glycosyltransferase Tox orthologs are relevant in gastrointestinal pathogens including *E. coli* and *C. difficile* where they contribute to virulence by disrupting epithelial barrier function (64). Strains harboring individual, chemically induced nonsense mutations in *tc0437*, *tc0438*, and *tc0439* were defective in GI colonization, but other genomic loci were associated with the defect in these highly mutagenized strains (65). GI-localized *Cm* does not appear to induce overt pathology (66). This observation seemingly contradicts a direct role of prototypical toxin activity, yet leaves open the possibility of colonization factor activity in that compartment. Further work addressing GI colonization in the absence of *tox* genes is needed to test these possibilities and further understand any potential link with our observation of oviduct pathology. This may also be relevant for human *Ctr* since emerging evidence indicates urogenital strains expressing truncated toxins may persist in the human GI tract (66). *Cm* has been used extensively to model genital tract infection in mice (67), and our data suggest that the effect of *tox* may be tissue-specific in that compartment, since *Cm* Δ*tox* exhibited different phenotypes from controls in the lower genital tract and oviduct, yet was indistinguishable from control strains in the uterine horn. It remains to be determined whether earlier resolution of lower genital tract infection might lead to faster clearance from the oviducts. The epithelial cells of the endocervix, endometrium, and oviduct differ from one another in function and morphology (68–70), and so investigation into the host cell-type-specific interactome of Tox proteins may reveal mechanistic insight into tissue-specific differences in burden and pathology. In the lower genital tract, minor differences in *Cm* Δ*tox* shedding did not correlate with equivalently high burdens in the upper genital tract, indicating that lower genital tract load of *Cm* Δ*tox* was sufficient for ascension through the cervical bottleneck and colonization of the upper genital tract. Bacterial burden in the upper genital tract, which was uniform across strains, also did not correlate with hydrosalpinx frequency, which was attenuated by the loss of *tox*. This is consistent with previous observations that *Cm* burden in the lower or upper genital tract does not significantly correlate with hydrosalpinx (70), nor does *Ctr* burden in the upper genital tract correlate with pathology (62). Whether *tox*-dependent differences in lower genital tract shedding impact oviduct-specific burden and/or transmission remains an interesting open question. Moreover, it remains to be determined whether the *Ctr tox* genes behave in a similar fashion as the *Cm tox* locus. *Ctr* D, containing *tox* gene *CT166,* induces robust oviduct pathology that is lacking after L2 infection despite equivalent upper genital tract burdens (62). If *Ctr tox* genes phenocopy those of *Cm*, efforts to inhibit Tox activity *in vivo* may result in decreases in shedding and/or oviduct pathology, making it an attractive candidate for future vaccines or therapeutics capable of reducing morbidity from *Chlamydia* infection in the female genital tract.

## MATERIALS AND METHODS

### Cell culture and organisms

These studies employed *C. muridarum* strain Nigg as the parent strain. *C. muridarum* Δ*tarp* (36) and *C. trachomatis* serovar L2 (LGV 434) were used as controls in some experiments. *E. coli* NEB-10β strain (New England Biolabs; NEB) was utilized in all cloning procedures. Chlamydiae were propagated where indicated in murine fibroblast McCoy cells (CRL-1696; ATCC) or human HeLa 229 epithelial cells (CCL-1.2, ATCC). Chlamydiae were purified using MD-76R (diatrizoate meglumine and diatrizoate sodium injection USP; Mallinckrodt Pharmaceuticals) as previously described (35). Unless otherwise noted, infections were accomplished by centrifugation at 900 × *g* for 60 min at 20°C. All cultures were maintained in RPMI 1640 containing 2 mM l-glutamine supplemented with 10% (vol/vol) heat-inactivated fetal bovine serum (Thermo Fisher) and incubated at 37°C in an atmosphere of 5% CO_2_ and 95% humidified air. Where indicated, media were supplemented with 0.6 ng/mL penicillin G, 2.5–15 ng/mL rifampin, or 50 µg/mL gentamicin sulfate.

*Cm* Δ*tox* was generated from WT *Cm* via FRAEM as described (36). The allelic exchange plasmid pOri-tox was generated by first amplifying 3 kb flanking regions surrounding *tc0437–tc0439*. The 5′ homology arm was PCR-amplified from *Cm* genomic DNA using Q5 high fidelity polymerase (NEB) and custom primers (Integrated DNA Technologies, IDT) 5armcytoMuri-F and 5armcytoMuri-R while the 3′ arm was amplified with primers 3armcytoMuri-F and 3armcytoMuri-F. PCR products were sequentially mobilized into pKW-CMori (36) using HIFI DNA Assembly Cloning Kit (NEB) to insert the arms into SalI (5′ arm) or SbfI (3′ arm) cut plasmid, respectively. DNA sequencing (Eurofins) was used to confirm fidelity of the resulting pKW-tox construct. Plasmid DNA was isolated from *E. coli dam*^−^/*dcm*^−^ (NEB) and used to transform *Chlamydia* using the CaCl_2_ method (71). Mutagenesis was accomplished to yield the *Cm* Δ*tox* strain as described (72) using the FRAEM protocol to allow allelic replacement of *tc0439–0439* with a *blaM-gfp* selection cassette. A Rifampin (Rif)-resistant derivative strain was generated as described previously (32) by first cultivating Δ*tox* for 4 passages in 2.5 ng/mL Rif, followed by 4 passages in 5 ng/mL Rif and final passage in 15 ng/mL Rif. Reversal of the tox deletion was accomplished by lateral gene transfer (32) via co-culture of Δtox^Rif^ with WT *Cm* at an initial 1:10 ratio in the absence of antibiotic selection for three 24 h passages. Subcultures were passaged twice in the presence of 15 ng/mL Rif. Six Rif-resistant, GFP-deficient (non-fluorescent) inclusions were physically picked using a 10 µL pipette tip and expanded using *tc0438*-specific qPCR to evaluate reversal. Two strains contained *tc0438* signal and designated as repaired (tox^Rep^). Clonal isolates were obtained for all strains as described by 2 sequential limiting dilution passages in 384 plates (73), and genomic manipulations were confirmed by whole-genome sequencing and variant analyses using *C. muridarum* Nigg AE002160.2 as reference (SeqCenter).

### DNA copy number and gene expression

Relative gene copy number was determined from McCoy cultures infected at a multiplicity of infection (MOI) of 1 with respective strains. DNA was extracted from 24 h cultures (74), and relative counts of *16S*, *tc0437*, *tc0438*, *tc0439*, or *gfp* were determined by quantitative real-time PCR using iTaq Universal SYBR Green Supermix (Bio-Rad). For assessment of gene expression, the Aurum Total RNA minikit (Bio Rad) was used to isolate RNA from McCoy cultures infected at an MOI of 1 for 24 h. Subsequent reverse-transcription PCR (RT-PCR) was accomplished using One*Taq* RT-PCR kit (NEB). For all reactions, specific elements were detected using custom (Integrated DNA technologies) gene-specific primers (Table S1), and amplification was accomplished using the CFX96 Real-Time System (Bio-Rad).

### Tissue culture infectivity

To assess intracellular growth, McCoy cells were seeded onto 6 well plates (2 × 10^5^ cells/well) 24 h prior to infection. Replicate wells were then infected either with WT, ∆*tox*, or tox^Rep^ *Cm*. To facilitate the infection, cultures were centrifuged at 900 × *g* for 1 h followed by an incubation at 37°C. Progeny EBs from 24 h cultures were passaged onto fresh McCoy and enumerated by immunofluorescence of cultures methanol fixed and stained with Hsp60 antibodies at 24 h. Invasion rates were tested by infection of McCoy monolayers on 12 mm glass coverslips at an MOI of 10 with respective strains. Infections were carried out in Hanks balanced salt solution (HBSS) by 900 × *g* centrifugation at 20°C for 8 min followed by incubation at 37°C for 30 min. All cultures were fixed with 4% paraformaldehyde at room temperature for 15 min and rinsed with phosphate-buffered saline (PBS). Extracellular EBs were labeled with a monoclonal antibody specific for chlamydial lipopolysaccharide (LPS) clone F70G (Invitrogen) followed by secondary antibody conjugated to Alexa 488. Samples were then permeabilized with 0.01% triton X100 and stained with whole-*Chlamydia*-specific rabbit antibodies followed by secondary antibody conjugated to Alexa 594. For each assay, the number of green (external) and red (total) EBs was determined over 10 fields of view. These data were then used to determine the percentage of internalized EBs. Data were collected from three individual replicate wells for each strain, and these percentages were then averaged together to give a final invasion rate. Finally, adherence of respective strains to host cells was tested. McCoy monolayers were mock-treated or treated with DEAE-dextran as described (35) 15 min prior to infection. Mock or dextran-treated monolayers were infected in triplicate by rocking at 37°C for 1 h with respective strains. Media were removed, and monolayers were thoroughly washed with HBSS. Whole-culture DNA was extracted, and chromosome copy number assessed via qPCR using 16s-specific primers (Table S1).

### Cytotoxicity assays

To assess cytotoxicity, McCoy cells were seeded onto 24 well plates with coverslips (1.5 × 10^5^ cells/well) 24 h prior to infection. Replicated wells were then mock infected or infected with *Ctr* L2 WT, *Cm* WT, *Cm Δtox*, or *Cm tox*^Rep^ at an MOI of 500. Cultures were centrifuged at 900 × *g* for 30 min followed by an incubation at 37°C. Four hours post infection, the cultures were fixed with 4% paraformaldehyde for 25 min and then permeabilized with ice-cold methanol for 5 min. Polymerized actin was detected using ActinRed phalloidin (ThermoFisher). Coverslips were washed with PBS and mounted. Epi-fluorescence microscopy was used to image coverslips using a 40× objective. ImageJ was used to process images. Freehand tool was used to outline the border of the cells, and the Measurement tool was used to measure circularity (4pi(area/perimeter^2^)). For this assay, 100 cells were measured for each triplicate for each strain. An LDH cytotoxicity assay was used to measure LDH release as an indicator of cytotoxicity. McCoy cells were seeded onto 96 well plates (2 × 10^4^ cells/well) 24 h prior to infection. Replicated wells were then mock infected or infected with *Ctr* L2 WT, *Cm* WT, *Cm* Δ*tox*, or *Cm* tox*^rep^* at an MOI of 500. Cultures were centrifuged at 900 × *g* for 30 min followed by an incubation at 37°C. Four hours post infection, 50 µL aliquots was taken from supernatant of cell culture and placed onto 96 well plate. LDH release was quantified using the CyQUANT LDH Cytotoxicity Assay Kit (Invitrogen). Cytotoxicity was calculated by comparing its LDH release in mock infected cells to the maximum LDH release (% cytotoxicity = ((sample LDH release- spontaneous LDH release)/(max LDH release-spontaneous LDH release) * 100). This percentage was then normalized to the chromosome copy number of the inoculum assessed via qPCR using 16s-specific primers (Relative cytotoxicity = % cytotoxicity/inoculum Ct values).

### Mice and genital tract infections

All mouse procedures were performed in accordance with protocols approved by the Institutional Animal Care and Use Committee of Mount Holyoke College (#BR-66-0624). Female C57BL/6 mice were purchased from The Jackson Laboratory (USA), housed at Mount Holyoke College, and provided food and water *ad libitum*. Intravaginal and transcervical infections were performed as has been previously described (62, 75). Briefly, 6- to 8-week-old female mice were treated s.c. with 2.5 mg depo-medroxyprogesterone acetate (Pfizer) 1 week before infection with density gradient purified EBs. Intravaginal infections were performed by clearing the vaginal vault with a calcium alginate tipped swab then instilling 10^5^ IFU of *C. muridarum* WT, Δ*tox,* or *tox*^Rep^ into the lower genital tract. Transcervical infections were performed using a nonsurgical embryo transfer device (ParaTechs) to instill 10^6^ IFU of *C. muridarum* WT, Δ*tox,* or *tox*^Rep^ directly into the upper genital tract. Infected mice were monitored for the duration of the study.

### Quantification of bacterial burden *in vivo*

To monitor lower genital tract shedding, vaginal swabs were collected at 3–28 days post infection, placed in 500 µL SPG, and vortexed with two glass beads for 5 min prior to freezing at −80°C. Serial dilutions were used to enumerate IFU using the tissue culture infectivity assay described above. To measure upper genital tract bacterial burden, qPCR was performed as previously described (62, 76) on total DNA isolated from homogenized upper genital tracts using a DNeasy Blood and Tissue Kit (Qiagen). *Chlamydia 16S* DNA and mouse *GAPDH* DNA were amplified and quantitated on an AriaMX Real-Time PCR System (Agilent) using specific primer pairs and probes (IDT or Applied Biosystems). The ratio of bacterial/mouse DNA in homogenates was calculated using standard curves generated from known amounts of purified DNA.

### Assessment of upper genital tract pathology

Gross pathology phenotypes (e.g., hydrosalpinx, uterine horn dilation) were evaluated *in situ* upon necropsy and imaged on a Canon EOS Rebel T3i camera immediately after excision of the female genital tract. Excised tissues were fixed in 10% formalin and processed by the Rodent Histopathology Core Facility at Harvard Medical School where they were embedded in paraffin, sectioned to 4–5 μm, then stained with H&E prior to being assessed for histopathology, as we previously described (62). In accordance with best practices in the literature (77, 78), each tissue was assigned a severity score based on the prevalence of pathology phenotypes relevant to human female upper genital tract infection with *C. trachomatis*, including fibrosis, edema, epithelial/membrane thickening and/or degeneration, luminal/tissue cellular infiltration, and increased vascularity. Left and right uterine horn, oviduct, and ovary were scored separately. Uterus pathology scores range from 0 (no pathology); 1 (mild/rare pathology, less than 1/3 of tissue affected); 2 (moderate/multifocal pathology, between 1/3 and 2/3 of tissue affected); to 3 (severe/coalescing pathology, greater than 2/3 of tissue affected). Oviduct and ovary scores ranged from 0 (no pathology), 1 (less than ½ the tissue affected), to 2 (more than ½ of the tissue affected). For chronic infection when oviduct edema can be extreme (hydrosalpinx visible by eye), histopathology was scored using an expanded 0–4 scale in the manner of Chen et al. (70) ranging from 0 (no pathology); 1 (less than ½ of the tissue affected), 2 (more than ½ of the tissue affected, though dilation is smaller than the ovary on the same side), 3 (more than ½ of the tissue affected with dilation equal to the ovary size on the same side), and 4 (more than ½ of the tissue affected with confluent dilation larger than the ovary on the same side). Two independent assessments were made for each tissue on randomized, anonymized slides, and if not in agreement, a third assessment was made. Inter-rating reliability was greater than 90%. Histopathology was imaged on a Nikon Eclipse 50i microscope with a PixeLink color camera. Cellsens and ImageJ software were used for acquisition, merged image acquisition for whole organ images, and linear adjustments to contrast and brightness as needed.

### Statistics

Statistical analyses were performed using GraphPad Prism (Version 6.04 or 10.4.1), are indicated in the appropriate figure legends, and were considered significant with a *P* value of < 0.05. Unless otherwise noted, tissue culture infectivity data are representatives from triplicate experiments where quantitative data were generated from experiments containing triplicate biological replicates. For animal studies, two to three independent experiments were performed for each analysis, with at least five mice per group per treatment.

## Data Availability

All source data associated with this report has been deposited in publicly available databases. Raw data files of imaged data and spreadsheets containing analyzed data are available at Mendeley Data (doi: 10.17632/wjh46bc5dh.1 and doi: 10.17632/byhjgdvbv7.1).

## SUPPLEMENTAL MATERIAL

Supplemental material iai.00419-25-s0001.docx Tables S1 and S2; Fig. S1 to S3.

ASM does not own the copyrights to Supplemental Material that may be linked to, or accessed through, an article. The authors have granted ASM a non-exclusive, world-wide license to publish the Supplemental Material files. Please contact the corresponding author directly for reuse.
