# Supplementary material for: Complete deletion of the Chlamydia muridarum putative cytotoxin locus reveals contributions during invasion in tissue culture and oviduct pathology during murine genital tract infection
Source: Infect Immun. 2025 Sep 22;93(10):e00419-25. doi: 10.1128/iai.00419-25 (PMC12519784; doi:10.1128/iai.00419-25)
Supplement: Supplemental material — Tables S1 and S2; Fig. S1 to S3. [file iai.00419-25-s0001.docx]

**Supplementary material**

**Table S1**. Primers used in this study.

| **Primer name** | **Primer Sequence (5’→ 3’)** |
| --- | --- |
| **Cloning Primers** |  |
| 5armcytoMuri-F | AACTCAAAATTTTACCACAGAAGTGCGATTTCTTTCCGAGCAATACCAGCTC |
| 5armcytoMuri-R | GCTATACGAAGTAGGGTCGAGATTCACCTTCACTATAGAATTATTTCTTTGTGTAA |
| 3armcytoMuri-F | TTATACGAAGTTATGACCTGCAAAAATTATGCACCAAAACTACTGCTTAAAACAAT |
| 3armcytoMuri-R | CGGGGTCTGACGCCCCGATAAAAGAAGCTCAGCGGCAC |
| **qPCR Primers^a^** |  |
| 16s-s | CCTGGTAGTCCTTGCCGTAAAC |
| 16s-as | TACTCCTCAGGCGGCATACTTA |
| tc0436-s | CTGAATAGAACTGGAGGCTTTCT |
| tc0436-as | CAAGCAGGAAGAGTGTGTAT |
| tc0437-s | CGGTGGCTATGCGTTCTTAT |
| tc0437-as | TGGCTTGCTCCACTACATTC |
| tc0438-s | TGGTTACAACATCGAGGGAATAG |
| tc0438-as | CCAATACACCAGGAACAGAAGA |
| tc0439-s | CGAAGCATCTCTGGCTTTACT |
| tc0439-as | CTGAAGGTCGTCCAAACTCTAC |
| tc0440-s | CTCTTCTTCTCGAGGACGTTTC |
| tc0440-as | GAAACTCAACGCCAATCCTTATC |
| rpoD-s | TGCAGCGTTGGATGGATAA |
| rpoD-as | CAGCCCTCGGTTGGTATATTT |
| tarp-s | CCGGAGGAATCATAGCTCATAC |
| tarp-as | CGCTGCATCATAGAGAGAGAC |
| gfp-s | GTGCCATGCCCGAAGGTTAT |
| gfp-as | CTTCAGCACGTGTCTTGTAGTTCC |

^a^ Primers were used for either qPCR (DNA) or qRT-PCR (RNA).

**Table S2.** SNP analysis of strains used in this study^a^

| **WT** | **Δtox** | **Δ*toxRif*** | ***tox-*Rep** | **Gene** | **position**^b^ | **mutation** | **putative function** |
| --- | --- | --- | --- | --- | --- | --- | --- |
| **X^c^** | **X** | **X** | **X** | intergenic | 126,408 | Δ1 bp | NA^d^ |
| **X** | **X** | **X** | **X** | intergenic | 126,417 | +A | NA |
| **X** | **X** | **X** | **X** | intergenic | 126,479 | A_5_→A_4_ | NA |
| **X** | **X** | **X** | **X** | *rlmD* | 141,411 | +A | RNA methyltransferase |
| **X** | **X** | **X** | **X** | *mraY* | 169,451 | +TTT | penta-peptide transferase |
| **X** | **X** | **X** | **X** | *rpmH* | 200,671 | G→C | 50S ribosomal protein L34 |
| **X** | **X** | **X** | **X** | intergenic | 403,623 | +AC | NA |
| **X** | **X** | **X** | **X** | *tc0341* | 403,652 | T→C | metal ABC transporter |
| **X** | **X** | **X** | **X** | *tc0342* | 404,885 | Δ1 bp | metal ABC transporter |
| **X** | **X** | **X** | **X** | *tc0408* | 468,392 | G→T | hypothetical protein |
|  | **X** | **X** | **X** | *tc0412* | 473,163 | Δ1 bp | inclusion membrane protein |
|  | **X** | **X** |  | *tc0437-4039* | 506484-536484 | deletion | cytotoxin locus |
| **X** |  |  | **X** | *tc0439* | 515,204 | C→G | putative cytotoxin |
| **X** | **X** | **X** | **X** | ubiX | 600,435 | C→A | flavin prenyltransferase |
|  |  | **X** | **X** | rpoB | 707,284 | A→G | RNA polymerase ß subunit |
| **X** | **X** | **X** | **X** | intergenic | 846,475 | Δ1 bp | NA |
| **X** | **X** | **X** | **X** | *omcB* | 866,121 | G→T | outer membrane protein |
| **X** | **X** | **X** | **X** | *tc0879* | 1,022,551 | G→A | hypothetical protein |

^a^ Variant analysis was performed using the *C. muridarum* Nigg annotated genome AE002160.2 as reference.

^b^ Genomic position of specific nucleotide change is indicated.

^C^ **X** denotes that the strain harbors the indicated polymorphism. Blank cells indicated the strain is unchanged at the locus compared to the reference genome.

^d^ NA = not applicable for changes outside of apparent coding sequences.


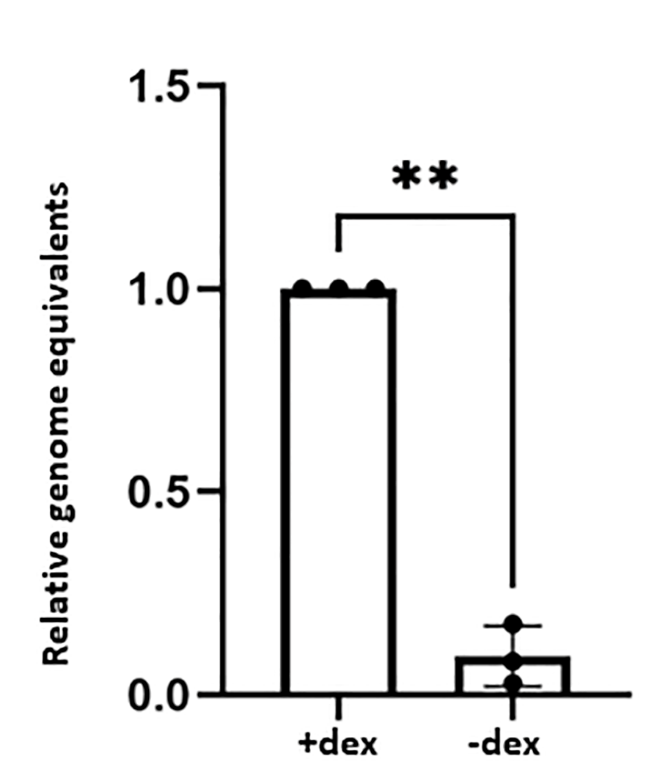


**Figure S1**. Dextran increases attachment efficiency. McCoy cells were left untreated (-dex) or pre-treated (+dex) with DEAE-Dextran and then equally infected with WT. Two hours post infection; monolayers were thoroughly washed with HBSS, and the attached bacteria were quantified by assessing chromosome copy number via qPCR. Data are represented as relative genome equivalents with the +dex treatment set to a value of 1.0. Standard deviations from triplicate samples are shown. Statistical significance was computed using Student’s T test with Welch’s correction (**, *P*<0.002).


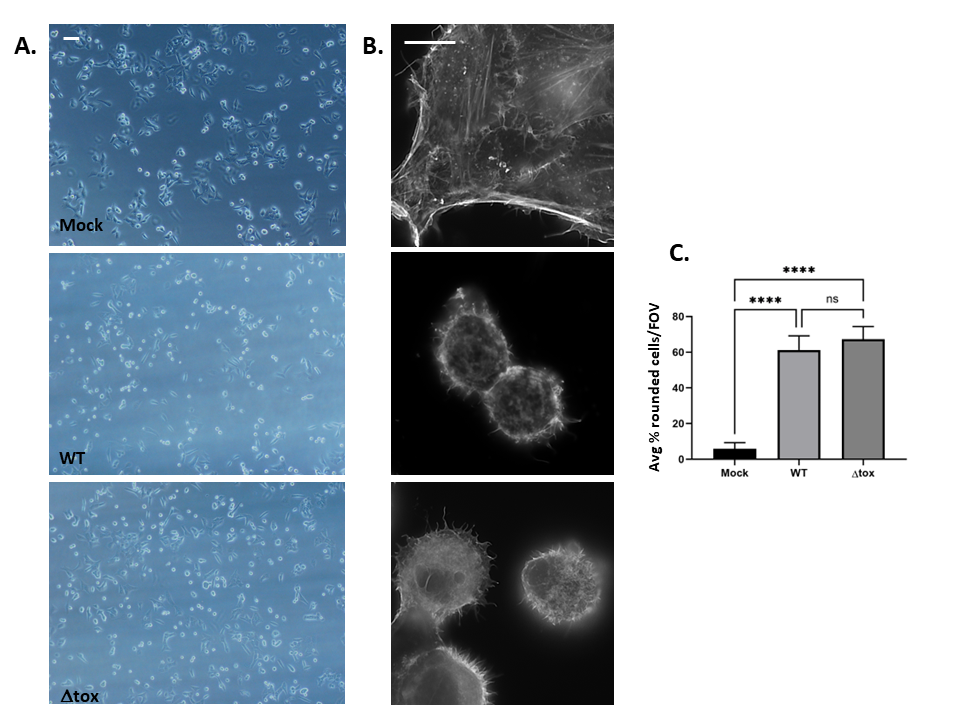


**Figure S2**. HeLa cells were mock infected or infected with WT or Δ*tox* *Cm* at an MOI of 200. Cultures were fixed at 3 hr post infection and imaged prior to fixation via light microscopy (A) then fixed with paraformaldehyde for epifluorescence visualization of phalloidin-stained actin localization (B). Representative fields of view are shown. Bars = 10 µm. (C). Cells were enumerated visually for 10 fields of view in light microscopy images. Data are presented as averages of percent rounded cells per field of view (FOV) for each strain. Statistical significance was evaluated by One-way ANOVA with multiple comparisons (ns = not significant, **; P < 0.0001).

**Figure S3. Ascension and gross pathology after intravaginal infection.** C57Bl/6 female mice were infected intravaginally with 10^5^ IFU of *C. muridarum* WT, Δ*tox,* or *tox*^Rep^. A) At 16 days post infection, ascension was measured by qPCR on upper genital tract homogenates. Data are presented as mean +/- SEM. Statistical significance was assessed by student’s t-test; ns: not significant. B) At 51 days post infection, female genital tracts were excised and assessed for gross pathology; asterisk indicates hydrosalpinx.
